# Hypofractionated and single-fraction radiosurgery for brain metastases with sex as a key predictor of overall survival

**DOI:** 10.1038/s41598-021-88070-5

**Published:** 2021-04-21

**Authors:** Julian Mangesius, Thomas Seppi, Katie Bates, Christoph R. Arnold, Danijela Minasch, Stephanie Mangesius, Johannes Kerschbaumer, Peter Lukas, Ute Ganswindt, Meinhard Nevinny-Stickel

**Affiliations:** 1 Department of Therapeutic Radiology and Oncology, Medical University of Innsbruck, Innsbruck, Austria; 2 Department for Medical Statistics, Medical University of Innsbruck, Innsbruck, Austria; 3 Department of Neuroradiology, Medical University of Innsbruck, Innsbruck, Austria; 4 Department of Neurosurgery, Medical University of Innsbruck, Innsbruck, Austria

**Keywords:** Radiotherapy, CNS cancer

## Abstract

Overall survival (OS) of patients with brain metastases treated with hypofractionated (HFSRT) or single-fraction (SRS) radiosurgery depends on several prognostic factors. The aim of this study was to investigate the potential of sex as an independent predictor of OS and evaluate the predictive accuracy of common prognostic scores. Retrospective analysis of 281 consecutive patients receiving radiosurgery of brain metastases was performed. Kaplan–Meier survival curves and Cox proportional hazards models were used to compare OS between SRS and HFSRT and by sex, before and after propensity-score matching (PSM) on key baseline prognostic covariates. Prognostic scores were evaluated using Harrell’s concordance index. Median OS was 11 months after both SRS and HFSRT. After PSM, median OS was 12 months after SRS (95% CI: 7.5–16.5) and 9 months after HFSRT (95% CI: 5.0–13.0; *p* = 0.77). Independent prognostic factors were sex, primary tumor, KPI, and systemic disease status. Median OS was 16 months for women and 7 months for male patients (*p* < 0.001). After excluding sex specific tumors, PSM revealed a median OS of 16 months for women and 8 months for male patients (*p* < 0.01). Evaluation of prognostic indices showed BSBM to be the most accurate (Harrell’s C = 0.68), followed by SIR (0.61), GPA (0.60), RPA (0.58), and Rades et al. (0.57). OS after HFSRT and SRS did not differ, although PSM revealed a non-significant advantage for SRS. Female sex was found to be a major independent positive prognostic factor for survival, and thus should be considered in the personalized decision-making of brain metastases treatment.

## Introduction

Oncologic disease leads to brain metastases in up to 40% of adult cancer patients. Treatment options include surgical resection, targeted systemic therapies, and stereotactic and/or whole brain irradiation^1^. Life expectancy depends on number, size, location, and origin of intracranial lesions. In addition, age, Karnofsky Performance Index (KPI), time to CNS metastases, extent of systemic disease, and status of primary disease have been identified as prognostic factors varyingly influencing overall survival (OS), and are thus included in several routinely applied prognostic indices. However, increasing evidence has been reported, that also sex is a predictor of survival in the majority of neoplastic diseases^2^. In addition, male sex has been identified as an independent risk factor for poor prognosis in metastatic patients mainly treated with extracranial stereotactic radiotherapy^3^. Whilst gender related-survival differences in patient cohorts with brain metastases have been reported sporadically, little importance has been attached to this aspect. Consequently, sex is not considered in the most common prognostic scores used to predict overall survival after stereotactic radiotherapy.

## Methods

### Data

Consecutive 281 patients underwent linear accelerator-based single fraction stereotactic radiosurgery (SRS) or hypofractionated stereotactic radiotherapy (HFSRT) between 08/2003 and 03/2018 without WBRT as initial therapy. All study procedures have been previously described^4^ and were carried out in accordance with relevant guidelines and Austrian regulations. The study was approved by the ethics committee of the Medical University of Innsbruck that waived the need of an informed consent for participants of retrospective studies.

Conformal SRS of retrospectively analyzed patients was applied with five to seven non-coplanar arcs. Planning-CT scans together with fused contrast-enhanced T1-weighed MRI scans were used to define target volumes. GTV was expanded by a margin of 1 mm for frameless radiotherapy (n = 228); no expansion was used for frame-based SRS (n = 127).

The median prescribed dose to the 80%-isodose was 20 Gy and ranged from 15 to 22 Gy for SRS. For HFSRT, applied dose concepts comprised 5 × 6.0 − 7.5 Gy, and 6 × 6.0 Gy prescribed to the 80%-isodose. Treatment modality and dose prescription were selected depending on metastasis-volume, location, proximity to vital structures, previous irradiation, and on tumor histology. PTV size varied between HFSRT and SRS (mean 3.1 ± 4.4 ccm vs. 1.6 ± 2.3 ccm). Cone-beam CT-verification was performed to correct patient position prior to treatment to < 1 mm xyz-translation, and < 1° rotation.

Follow-up was performed with clinical examination and contrast enhanced MRI scans every 3 months until death (up to 118 months). Elapsed time from first SRS-treatment until death was defined as overall survival. Surviving patients at the end of the study were censored at the day of last follow-up (n = 45, 16%).

### Statistical analysis

Propensity scores were calculated using multivariable logistic regression with the covariates age, sex, primary tumor, number of CNS metastases, PTV volume, KPI, presence of extracerebral metastases, and systemic disease status to determine the conditional probability of the patient receiving SRS vs. HFSRT, using 1:1 matching without replacement. A Kaplan–Meier analysis was conducted to estimate differences in survival by mode for all fully documented cases in the cohort (complete case analysis, n = 274) and among the matched pairs population (n = 168). Univariate Cox proportional hazards models were performed for OS for all presumed prognostic factors. Multivariable Cox proportional hazard models were performed adjusted for prognostic factors determined by backwards stepwise selection.

To assess gender related differences in survival, Kaplan–Meier curves were fitted and the log-rank test performed on all patients (n = 274), as well as among all patients excluding those with breast cancer as a primary tumor type (n = 240). Propensity-score matching for sex was also performed considering age, primary tumor, number of CNS metastases, PTV volume, KPI, presence of extracerebral metastases, and systemic disease status using 1:1 matching without replacement and excluding patients with breast cancer as the primary tumor type. A Kaplan–Meier survival curve and log-rank test was conducted on the matched pair sample (n = 164).

The prognostic indices (PI) RPA (Recursive Partitioning Analysis)^5^, GPA (Graded Prognostic Assessment)^6^, SIR (Score Index For Radiosurgery)^7^, BSBM (Basic Score for Brain Metastases)^8^, and Rades et al.^9^ were calculated (see supplementary Table 1) and their predictive accuracy of survival was evaluated using Harrell’s concordance index (Harell’s C). Data (all available cases, n = 265) were split randomly into training and test subsets in Stata/MP 15.1. Separate Cox models were run for each prognostic score using the training dataset with Harrell’s C estimates for the test subset. Harrell’s C can take a value between 0 and 1; a value of 1 corresponds to perfect concordance whilst a value of 0.5 indicates completely random concordance. Pairwise comparisons of Harrell’s C were conducted using the RPA index as the reference.

### Ethics approval

This study has been approved by the research ethics committee of the Medical University Innsbruck (Reference Number: EK 1160/2018).

## Results

### Overall survival and treatment technique

**Figure 1:**
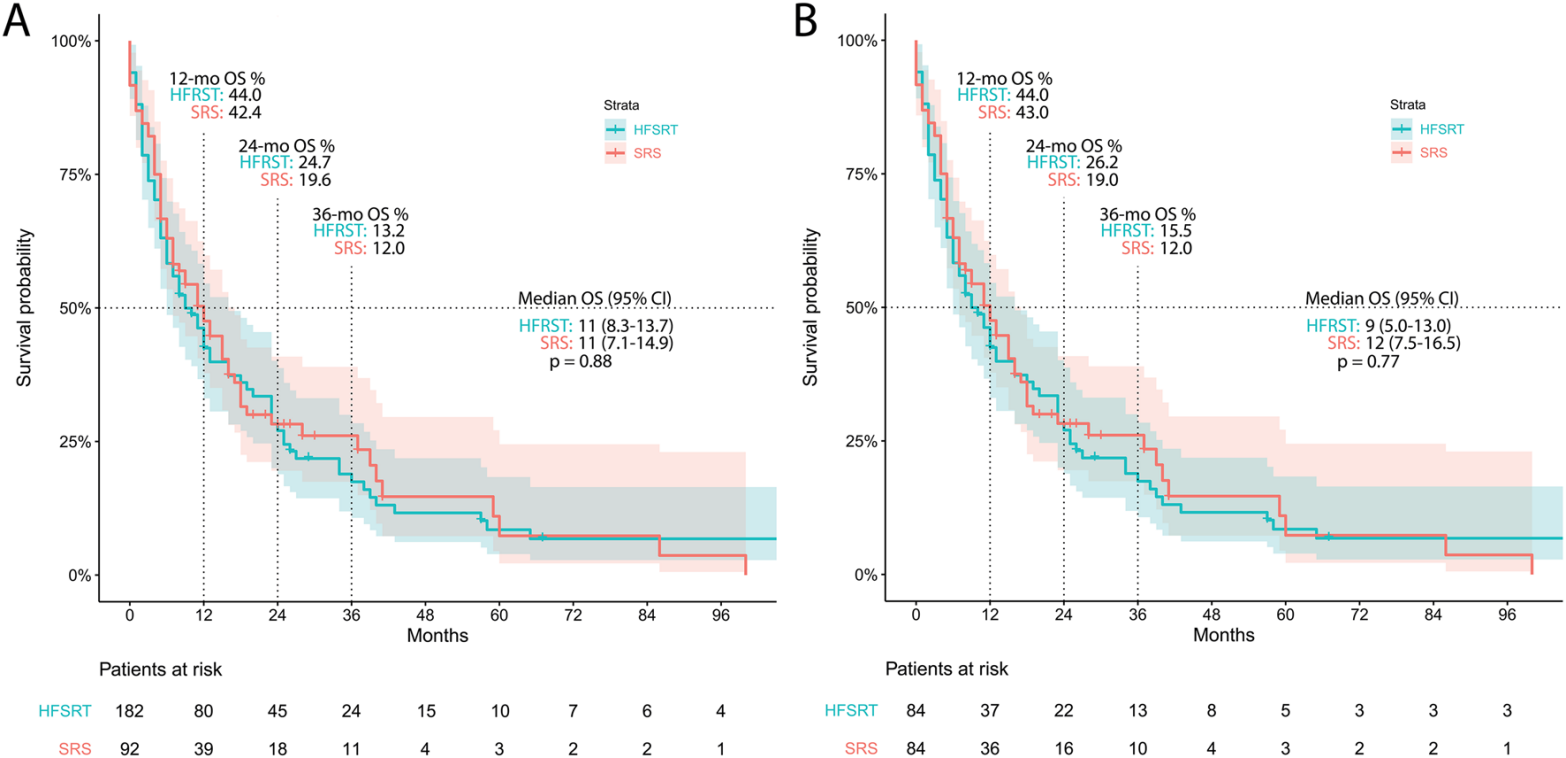
Kaplan–Meier analysis of survival by treatment technique before (**A**) and after (**B**) propensity score matching.

Patient characteristics are displayed in Table 1. Summative median OS, independent of treatment and patient characteristics (n = 274), was 11 months (95% CI: 8.8–13.2 months). Overall Survival of patients treated with HFSRT (n = 182) was 11 months (95% CI: 8.3–13.7 months), and OS of SRS treated patients (n = 92) was 11 months (95% CI: 7.1–14.9; Fig. 1A).

**Table 1:** Patient characteristics.

	HFSRT	SRS	Male	Female
**Patients**	182	92	148	126
**Age (years)**				
Mean (SD)	61.5 (11.0)	60.5 (12.6)	63.3 (10.4)	58.7 (12.4)
Median (IQR)	63 (54–70)	62.5 (52–70)	64 (56–70.5)	61 (49–69)
**Sex**				
Female	85 (46.7%)	41 (44.4%)		
Male	97 (53.3%)	51 (55.4%)		
**Primary tumor**				
Lung adeno	84 (46.2%)	34 (37.0%)	68 (46.0%)	50 (39.7%)
Lung Squamous	17 (9.3%)	7 (7.6%)	18 (12.2%)	6 (4.8%)
Breast	24 (13.2%)	10 (10.9%)	0 (0.0%)	34 (27.0%)
Melanoma	22 (12.1%)	17 (18.5%)	22 (14.9%)	17 (13.5%)
Kidney	7 (3.9%)	8 (8.7%)	12 (8.1%)	3 (2.4%)
Other	28 (15.4%)	16 (17.4%)	28 (18.9%)	16 (12.7%)
**Number of metastases treated (lifetime)**				
Median (IQR)	1 (1–2)	1 (1–3)	1 (1–2)	1 (1–2)
Mean (SD)	1.8 (1.2)	1.9 (1.1)	1.8 (1.2)	1.8 (1.2)
**Time to CNS metastasis (months)**				
Mean (SD)	29.5 (46.3)	34.6 (53.7)	27.3 (41.0)	35.8 (56.5)
Median (range)	13.5 (4–33)	14 (0–43)	12 (0–36)	15 (5–38)
**Dose HFSRT**				
5 × 6	23 (12.6%)		9 (6.1%)	14 (11.1%)
5 × 7	85 (46.7%)		48 (32.4%)	37 (29.4%)
6 × 6	10 (5.5%)		5 (3.4%)	5 (4.0%)
5 × 7.5	64 (35.2%)		35 (23.7%)	29 (23.0%)
**Dose SRS**				
1 × 20		64 (69.6%)	39 (26.4%)	25 (19.8%)
1 × 18		21 (22.8%)	10 (6.8%)	11 (8.7%)
1 × 15		3 (3.3%)	0 (0%)	3 (2.4%)
1 × 22		4 (4.4%)	2 (1.4%)	2 (1.6%)
**KPI**				
Mean (SD)	8.6 (1.2)	8.6 (1.5)	8.5 (1.3)	8.7 (1.3)
Median (IQR)	9 (8–10)	9 (8–10)	9 (8–10)	9 (8–10)
**Smoking status**				
Active	65 (35.7%)	30 (32.6%)	53 (35.8%)	42 (33.3%)
Former	27 (14.8%)	21 (22.8%)	33 (22.3%)	15 (11.9%)
Never	78 (42.9%)	30 (32.6%)	51 (34.5%)	57 (45.2%)
Unknown	12 (6.6%)	11 (12.0%)	11 (7.4%)	12 (9.5%)
**Extracerebral metastases**				
Not present	47 (25.8%)	19 (20.7%)	35 (23.7%)	31 (24.6%)
Present	135 (74.2%)	73 (79.3%)	113 (76.3%)	95 (75.4%)
**Systemic disease status**				
Controlled	77 (42.3%)	37 (40.2%)	55 (37.2%)	59 (46.8%)
Progressive	105 (57.7%)	55 (59.8%)	93 (62.8%)	67 (53.2%)
**RPA class**				
I	20 (11.0%)	7 (7.6%)	12 (8.1%)	15 (11.9%)
II	156 (85.7%)	78 (84.8%)	130 (87.8%)	104 (82.5%)
III	6 (3.3%)	7 (7.6%)	6 (4.1%)	7 (5.6%)
**Number of RT**	228	127	191	164
**Number of RTs per patient**				
Median (range)	1 (1–5)	1 (1–4)	1 (1–5)	1 (1–4)
Mean	1.3 (0.7)	1.2 (0.6)	1.3 (0.7)	1.3 (0.7)
**Number of metastases**	331	207	283	255
**Number of metastases per RT**				
Median (range)	1 (1–5)	1 (1–5)	1 (1–5)	1 (1–5)
IQR	1–2	1–2	1–2	1–2
Mean	1.45	1.63	1.48	1.55
**PTV volume (ccm)**				
Mean (SD)	3.1 (4.4)	1.6 (2.3)	2.6 (3.7)	2.4 (3.7)
Median (range)	1.49 (0.11–31.54	0.53 (0.04–13.3)	1.26 (0.04–31.5)	1.0 (0.04–30.7)
IQR	0.65–3.51	0.19–1.98	0.52–2.98	0.31–2.85

After propensity matching (n = 84 pairs) to improve homogeneity of prognostic factor variance between patients treated with HFSRT or SRS (see Supplementary Table 2 and 3), OS was larger among patients treated with SRS, although this did not differ significantly (*p* = 0.77; median OS_HFSRT_ = 9 months, 95% CI: 5.0–13.0 months; median OS_SRS_ = 12 months, 95% CI 7.5–16.5 months; Fig. 1B).

### Prognostic factors

**Figure 2:**
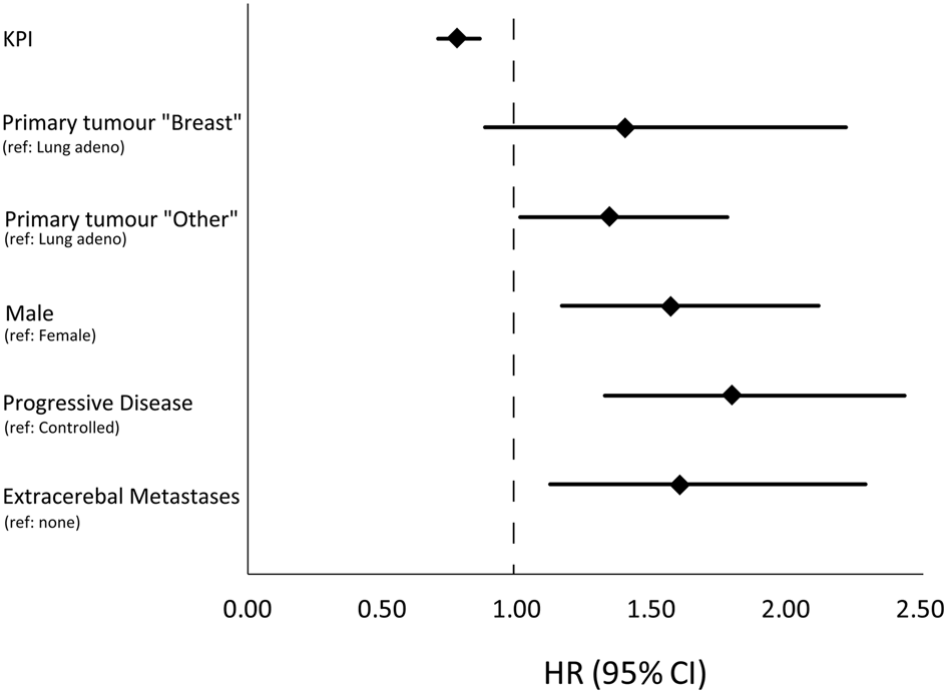
Multivariate analysis of significant prognostic factors for survival.

Univariate analysis of prognostic factors showed poor survival outcomes (n = 274) to be associated with higher age, male sex, lower KPI, primary tumor type, existence of extracerebral metastases, and progressive systemic disease status. On multivariate analysis (Fig. 2), impaired OS was confirmed to be independently associated only with sex (HR = 1.57, 95% CI: 1.17–2.12), primary tumor (other vs. lung; HR = 1.35, 95% CI: 1.01–1.79), KPI (HR = 0.78, 95% CI: 0.71–0.86), and systemic disease status (HR = 1.61, 95% CI: 1.12–2.30). PTV size did not significantly influence OS in both analyses.

**Figure 3:**
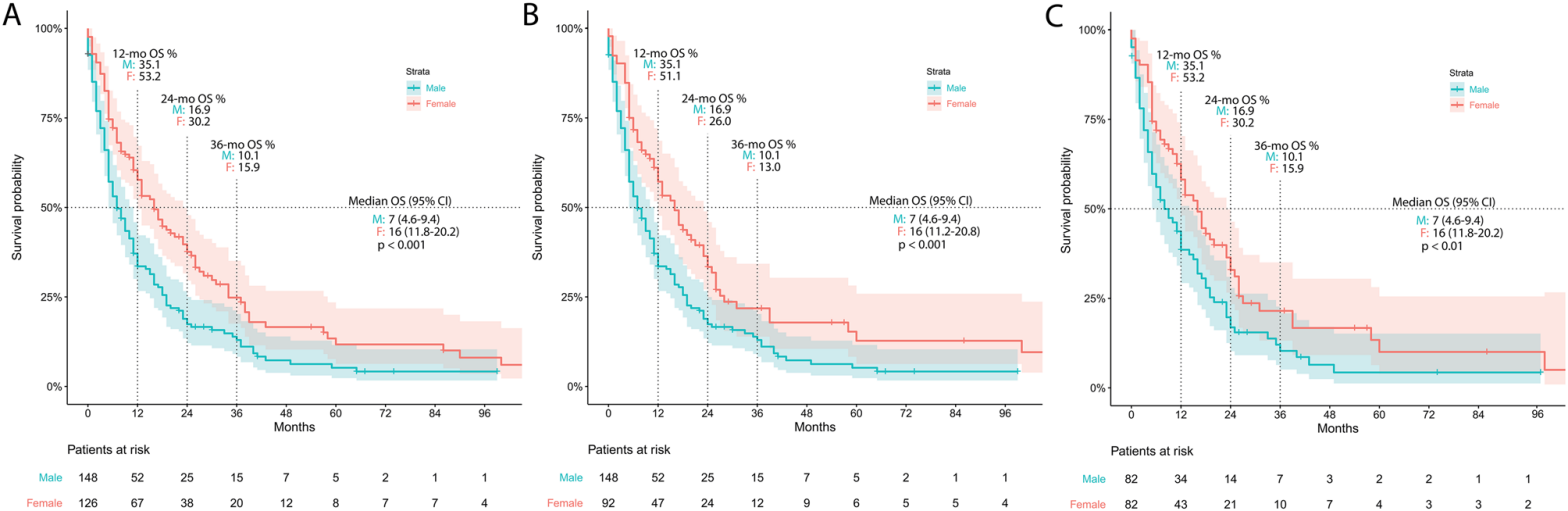
Kaplan–Meier analysis of survival by sex with (**A**) and without (**B**) inclusion of breast cancer patients, and after propensity score matching for prognostic factors other than sex (**C**).

Female patients in our cohort (n = 126 women versus 148 men) had a markedly prolonged survival (*p* < 0.001, median OS_female_ = 16 months, 95% CI: 11.8–20.2 months; median OS_male_ = 7 months, 95% CI: 4.6–9.4 months; Fig. 3A). After exclusion of patients with breast cancer, sex related OS was 16 months for female patients (n = 92; 95% CI: 11.2–20.8 months) and 7 months for men (n = 148; 95% CI: 4.6–9.4 months), still evidencing a highly significant difference in OS (*p* = 0.001—Fig. 3B). In the balanced propensity matched pair cohort (n = 82 pairs), female patients preserve a two-fold higher median OS compared to males (*p* = 0.009; median OS_female_ = 16 months, 95%CI: 11.8–20.2 months; median OS_male_ = 8 months, 95%CI: 3.6–12.4 months; Fig. 3C).

Smoking status was available for 251 (92%) of 274 patients (Table 1). After propensity score matching, the distribution presented as follows: 35.6% of male and 32.8% of female patients were active smokers, 22.1% of male and 11.7% of female patients were former smokers, and 34.2% of male and 44.5% of female patients were never smokers.

### Comparison of prognostic scores among patients with brain metastases

**Figure 4:**
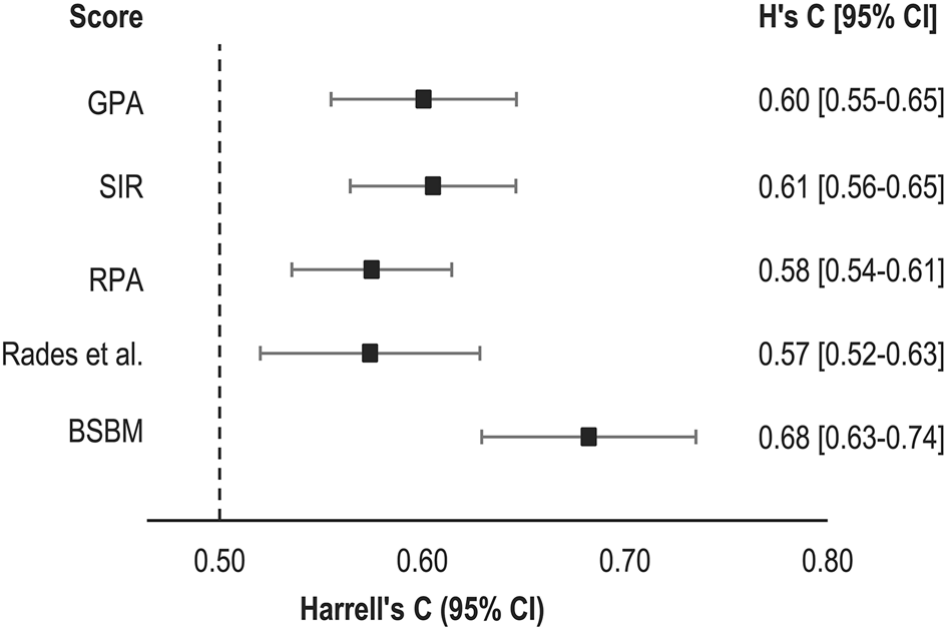
Comparison of prognostic indices using Harrell’s C.

For all prognostic scores, Harrell’s Cs were significantly larger than 0.5, although none surpass a value of 0.7 and all were accompanied by wide confidence intervals (Fig. 4). Pairwise comparisons indicate that BSBM predicts survival better than the most commonly used RPA (Table 2); training dataset models are available in Supplementary Table 4.

**Table 2:** Pairwise comparisons of Harrell’s C to RPA.

	Difference in H’s C	*p* value	LB	UB
BSBM	− 0.11	< 0.001	− 0.16	− 0.06
Rades et al	0.00	n.s	− 0.05	0.05
SIR	− 0.03	n.s	− 0.08	0.02
GPA	− 0.03	n.s	− 0.07	0.02

## Discussion

### Overall survival and treatment technique

With long term follow-up of up to 118 months after first radiosurgery, we report about a summative median OS of 11 months after both HFSRT (n = 182) and SRS (n = 92) of limited brain metastasis (1–5). One- and two-year OS was 44.6% and 26.4%, respectively. Thus, our data adds to the previously published literature that reports about OS ranging from 6 to 17 months for patients with brain metastases of various size, number, and histology after HFSRT and SRS^10–25^.

Most of our patients were treated with an envisaged Biologically Effective Dose (BED) (at α/β = 10) of 60 Gy, thus dose concepts were comparable to previously published series of HFSRT and SRS applications^19,22,23^.

As frequently reported in literature, patient cohorts receiving HFSRT and SRS differ substantially in number and size of metastases, location and histology, as well as in other prognostic factors. Conversely to SRS, HFSRT is often favored in patients with large metastases or lesions located in eloquent brain areas^19,25^. Therefore, comparative analysis suffers from differing dose concepts and inhomogeneous patient characteristics because of unequal allocation to the two treatment modalities. In order to minimize this effect we performed a propensity score matching to pair each SRS patient with a best matching HFSRT patient, including all relevant prognostic factors for pairing. As a result, homogeneity in the distribution of prognostic factors between patients treated with SRS and HFSRT improved significantly, thereby enabling unbiased direct comparison. Survival analysis after PSM showed a numerical advantage for SRS over HFSRT (12 vs. 9 months), which does not, however, translate to significance (*p* = 0.77). The median OS observed for SRS after PSM was 3 months longer than for HFSRT, but confidence intervals of both cohorts were strongly overlapping (HFSRT: 5.0–13.0 months, SRS: 7.5–16.5 months). Considering also the sizable number of 84 matching pairs, the absence of statistical significance regarding the numerical survival advantage of SRS reflects the clinical evidence of HFSRT being on par with SRS^22,26^.

### Prognostic factors and scores

In the era of personalized medicine, individualized estimation of survival time becomes more and more important, since therapy decision hinges on survival expectation. In order to detect patients who are either at risk for early death or who have a chance for long-term survival, inclusion and specific weighting of prognostic factors are decisive. Thus, independent factors have been identified in the past, including age, KPI, primary tumor, presence of extracranial metastases, number and size of intracranial metastases, and systemic disease status. These prognostic factors have been condensed into various prognostic scores of differing clinical applicability, reliability and prognostic value. The major weakness of all established scores to predict OS are the facts that they do not deliver individualized survival probabilities and that they tend to suffer from disproportionally sized prognostic groups^27^. Collaterally, and despite continuous validation of prognostic scores in use for decades, ongoing improvement in cancer therapy may lead to outdated survival estimations. Our results confirm higher age, male sex, lower KPI, primary tumor type other than lung adenocarcinoma or breast cancer, existence of extracerebral metastases, and progressive systemic disease status as to represent hazardous factors for survival. However, multivariate analysis of our data revealed that only sex, primary tumor, KPI, and systemic disease status are independently influencing OS. Male sex and progressive systemic disease status revealed to equally contribute to the hazard of poor survival (HR: 1.5 vs. 1.6), both identified as the strongest predictors of unfavorable outcome in our cohort of 274 patients.

Gender-related differences in OS of patients with brain metastases occasionally have been reported in the past also for large patient cohorts^27–29^. However, there exist also competing results derived from multicenter studies, which did not identify gender related differences in OS. For instance, Sperduto et al. established the prognostic GPA score by analyzing five cohorts from randomized clinical trials (1,960 patients in total) in 2011. A significant gender effect on patient outcome was not observed. Consequently, sex has not been considered in the meanwhile widely used GPA score or in other prognostic indices. However, the GPA and similar scores^5,9^ are based on patient cohorts of which only a minority was treated by radiosurgery. In addition, median overall survival of 75% of the investigated patients by Sperduto et al. did not exceed 3.8 months^6^. By contrast, in our study including only patients treated by radiosurgery, median survival of the entire cohort was 11 months, and we clearly identified gender as an independent prognostic factor for our cohort. A potential gender related difference might only become evident in patients of better prognosis and increased overall survival. This is supported by the fact that female survival advantages were primarily observed in newer patient cohorts benefitting from increased utilization of targeted therapies and radiosurgery.

In order to prove the independency and validity of sex as an underestimated factor of OS prognosis, exclusion of patients afflicted by sex specific primaries and balancing the distribution of prognostic factors among male and female patients by propensity matching were performed in statistical evaluation. Even after unbiased comparison, median OS of female patients (16 months) was still twice the median OS of male patients (8 months). We therefore conclude that a favorable distribution of other decisive hazard parameters, such as the smoking status, among the female cohort can, if at all, only partially explain the pronounced survival advantage. Thus, it is very likely that sex itself or other unknown gender related characteristics of female patients might be responsible for the observed effect. Female survival advantages have been reported for a range of oncologic diseases. Sex differences have been observed in the genetic and molecular basis of cancer, and the efficacy and toxicity of anticancer therapy^30^. Especially in the treatment of lung cancer, the main origin of brain metastases, several molecular markers such as EGFR, ALK, or PD1 can be exploited for targeted therapy approaches. An increased benefit of women has been observed for EGFR inhibitors^31^. Conversely, men profit from treatment with immune checkpoint inhibitors^31,32^. The contribution of such medication on the observed gender differences in the outcome of brain metastases treatment by stereotactic radiosurgery however has still to be investigated. Irrespective of benefits for female patients that derive from pharmacogenomic differences between the sexes, the observed survival advantage of female patients with brain metastases in our cohort is valid for both applied treatment options, HSFRT and SRS.

Validation of common prognostic scores applied to our patient cohort (Fig. 4) evidenced BSBM as to best perform regarding subgroup discrimination and OS prediction. After further evaluation of the observed gender effect, sex could be included as a parameter in existing scores such as the BSBM, thereby potentially increasing their prediction accuracy. Our work reinforces the necessity to continuously adjust and reevaluate prognostic scores in use in clinical practice, especially if applied to patients with brain metastases treated with rapidly evolving radiotherapy methodologies and systemic therapies.

## Conclusion

After long-term follow-up, no difference in overall survival between single-fraction and hypofractionated radiosurgery of brain metastases could be observed in our cohort of 274 consecutive patients. Female sex turned out to be a major independent prognostic factor for overall survival, only in part explainable by a more favorable distribution of other hazard parameters, if compared to the male cohort. Population-based studies or meta-analyses should be conducted to validate our findings and to encourage discussion for the inclusion of sex and gender characteristics in the decision-making for the personalized treatment of patients with brain metastases.

## Supplementary Information

Supplementary Tables.

## Data Availability

The datasets used and analysed during the current study are available from the corresponding author on reasonable request.

## References

[1] Tsao MN (2012). Radiotherapeutic and surgical management for newly diagnosed brain metastasis(es): An American Society for Radiation Oncology evidence-based guideline. Pract. Radiat. Oncol..

[2] Jung KW (2012). Do female cancer patients display better survival rates compared with males? Analysis of the Korean National Registry data, 2005–2009. PLoS ONE.

[3] Van den Begin R (2019). The METABANK score: A clinical tool to predict survival after stereotactic radiotherapy for oligometastatic disease. Radiother. Oncol..

[4] Mangesius, J. *et al.* Hypofractionated and single-fraction radiosurgery for brain metastases with sex as a key predictor of overall survival. 10.21203/rs.3.rs-41042/v1 (2020). 10.1038/s41598-021-88070-5 PMC8060341 33883632

[5] Gaspar L (1997). Recursive partitioning analysis (RPA) of prognostic factors in three Radiation Therapy Oncology Group (RTOG) brain metastases trials. Int. J. Radiat. Oncol. Biol. Phys..

[6] Sperduto PW, Berkey B, Gaspar LE, Mehta M, Curran W (2008). A new prognostic index and comparison to three other indices for patients with brain metastases: An analysis of 1,960 patients in the RTOG database. Int. J. Radiat. Oncol. Biol. Phys..

[7] Weltman E (2000). Radiosurgery for brain metastases: A score index for predicting prognosis. Int. J. Radiat. Oncol. Biol. Phys..

[8] Lorenzoni J (2004). Radiosurgery for treatment of brain metastases: Estimation of patient eligibility using three stratification systems. Int. J. Radiat. Oncol. Biol. Phys..

[9] Rades D, Dunst J, Schild SE (2008). A new scoring system to predicting the survival of patients treated with whole-brain radiotherapy for brain metastases. Strahlenther. Onkol..

[10] Kocher M (2011). Adjuvant whole-brain radiotherapy versus observation after radiosurgery or surgical resection of one to three cerebral metastases: Results of the EORTC 22952–26001 study. J. Clin. Oncol..

[11] Rades D (2007). Whole-brain radiotherapy versus stereotactic radiosurgery for patients in recursive partitioning analysis classes 1 and 2 with 1 to 3 brain metastases. Cancer.

[12] Wang LG (2002). Brain metastasis: Experience of the Xi-Jing hospital. Stereotact. Funct. Neurosurg..

[13] Li B (2000). Comparison of three treatment options for single brain metastasis from lung cancer. Int. J. Cancer.

[14] Aoyama H (2006). Stereotactic radiosurgery plus whole-brain radiation therapy vs stereotactic radiosurgery alone for treatment of brain metastases: A randomized controlled trial. JAMA.

[15] Chang EL (2009). Neurocognition in patients with brain metastases treated with radiosurgery or radiosurgery plus whole-brain irradiation: A randomised controlled trial. Lancet Oncol..

[16] Brown PD (2016). Effect of radiosurgery alone vs radiosurgery with whole brain radiation therapy on cognitive function in patients with 1 to 3 brain metastases: A randomized clinical trial. JAMA.

[17] Yamamoto M (2017). A multi-institutional prospective observational study of stereotactic radiosurgery for patients with multiple brain metastases (JLGK0901 study update): Irradiation-related complications and long-term maintenance of mini-mental state examination scores. Int. J. Radiat. Oncol. Biol. Phys..

[18] Lockney NA (2017). Clinical outcomes of patients with limited brain metastases treated with hypofractionated (5x6Gy) conformal radiotherapy. Radiother. Oncol..

[19] Aoyama H (2003). Hypofractionated stereotactic radiotherapy alone without whole-brain irradiation for patients with solitary and oligo brain metastasis using noninvasive fixation of the skull. Int. J. Radiat. Oncol. Biol. Phys..

[20] Aoki M, Abe Y, Hatayama Y, Kondo H, Basaki K (2006). Clinical outcome of hypofractionated conventional conformation radiotherapy for patients with single and no more than three metastatic brain tumors, with noninvasive fixation of the skull without whole brain irradiation. Int. J. Radiat. Oncol. Biol. Phys..

[21] Matsuyama T, Kogo K, Oya N (2013). Clinical outcomes of biological effective dose-based fractionated stereotactic radiation therapy for metastatic brain tumors from non-small cell lung cancer. Int. J. Radiat. Oncol. Biol. Phys..

[22] Kim YJ (2011). Single-dose versus fractionated stereotactic radiotherapy for brain metastases. Int. J. Radiat. Oncol. Biol. Phys..

[23] Fokas E (2012). Stereotactic radiosurgery and fractionated stereotactic radiotherapy: Comparison of efficacy and toxicity in 260 patients with brain metastases. J. Neurooncol..

[24] Rajakesari S (2014). Local control after fractionated stereotactic radiation therapy for brain metastases. J. Neurooncol..

[25] Minniti G (2014). Fractionated stereotactic radiosurgery for patients with brain metastases. J. Neurooncol..

[26] Minniti G (2016). Single-fraction versus multifraction (3 x 9 Gy) stereotactic radiosurgery for large (>2 cm) brain metastases: A comparative analysis of local control and risk of radiation-induced brain necrosis. Int. J. Radiat. Oncol. Biol. Phys..

[27] Zindler JD (2017). Individualized early death and long-term survival prediction after stereotactic radiosurgery for brain metastases of non-small cell lung cancer: Two externally validated nomograms. Radiother. Oncol..

[28] Yamamoto M (2014). Stereotactic radiosurgery for patients with multiple brain metastases (JLGK0901): A multi-institutional prospective observational study. Lancet Oncol..

[29] Minniti G (2011). Stereotactic radiosurgery for brain metastases: Analysis of outcome and risk of brain radionecrosis. Radiat. Oncol..

[30] Kim HI, Lim H, Moon A (2018). Sex differences in cancer: Epidemiology, genetics and therapy. Biomol. Ther. (Seoul).

[31] Pinto JA (2018). Gender and outcomes in non-small cell lung cancer: An old prognostic variable comes back for targeted therapy and immunotherapy?. ESMO Open.

[32] Wang S, Cowley LA, Liu X-S (2019). Sex differences in cancer immunotherapy efficacy, biomarkers, and therapeutic strategy. Molecules.

