# Supplementary material for: Hypofractionated and single-fraction radiosurgery for brain metastases with sex as a key predictor of overall survival
Source: Sci Rep. 2021 Apr 21;11:8639. doi: 10.1038/s41598-021-88070-5 (PMC8060341; doi:10.1038/s41598-021-88070-5)
Supplement: Supplementary file 1 — Supplementary Tables. [file 41598_2021_88070_MOESM1_ESM.docx]

Supplementary Material

Hypofractionated and single-fraction radiosurgery for brain metastases with sex as a key predictor of overall survival

# Authors:

Julian, Mangesius, MD (1), Julian.mangesius@i-med.ac.at

Thomas, Seppi, PhD (1), thomas.seppi@i-med.ac.at

Katie, Bates, PhD (2), katie.bates@i-med.ac.at

*Christoph Reinhold, Arnold, MD, PhD (1), christoph.arnold@i-med.ac.at

Danijela, Minasch, MD (1), danijela.minasch@i-med.ac.at

Stephanie, Mangesius, MD, PhD (3), stephanie.mangesius@i-med.ac.at,

Johannes, Kerschbaumer, MD, PhD (4), j.kerschbaumer@i-med.ac.at

Peter, Lukas MD (1), peter.lukas@i-med.ac.at

Ute, Ganswindt, MD, PhD (1), ute.ganswindt@i-med.ac.at

Meinhard, Nevinny-Stickel, MD, PhD (1), Meinhard.Nevinny@i-med.ac.at

(1) Department of Therapeutic Radiology and Oncology, Medical University of Innsbruck, Austria
(2) Department for Medical Statistics, Medical University of Innsbruck, Austria
(3) Department of Neuroradiology, Medical University of Innsbruck, Austria
(4) Department of Neurosurgery, Medical University of Innsbruck, Innsbruck, Austria

**Corresponding author**

Arnold Christoph Reinhold
e-mail: christoph.arnold@i-med.ac.at
telephone: +43 50 504 82917
address: Univ.-Klinik für Strahlentherapie-Radioonkologie, Anichstrasse 35, A-6020 Innsbruck, Austria

**Supplementary table 1: Overview of prognostic indices used in this study.**
NED: no evidence of disease, CR: complete remission. Time to CNS treatment: time from diagnosis to treatment of cerebral metastases

|  | RPA (Gaspar et al., 1997) | GPA (Sperduto et al., 2008) | SIR (Weltman et al., 2000) | BSBM (Lorenzoni et al., 2004) | Rades et al., 2008 |
| --- | --- | --- | --- | --- | --- |
| KPS | >70 | <70: 0 70-80: 0.5 >80: 1 | <60: 0 60-70: 1 >70: 2 | ≤70: 0 >70: 1 | <70: 1 ≥70: 5 |
| Age | <65 | >60: 0 50-90: 0.5 <50: 1 | ≥60: 0 51-59: 1 ≤50: 2 |  | >60: 3 ≤60: 4 |
| Systemic disease |  |  | progressive: 0 stable: 1 CR or NED |  |  |
| Primary tumor | controlled |  |  | not controlled: 0 controlled: 1 |  |
| Extracranial metastases | not present | present: 0 not present: 1 |  | present: 0 not present: 1 | present or uncontrolled primary tumor: 2 not present: 5 |
| Number of CNS metastases |  | >3: 0 2-3: 0.5 1: 1 | ≥3: 0 2: 1 1: 2 |  |  |
| Volume of largest lesion |  |  | >13: 0 5-13: 1 <5: 2 |  |  |
| Time to CNS treatment |  |  |  |  | ≤ 8 months: 3 > 8 months: 4 |
| Classes: | I: all criteria above met II: all others III: KPS <70 | III: 0-1.0 points II: 1.5-2.5 points I: 3.5-4.0 points | III:0-3 points II: 4-7 points I:8-10 points | IV: 0 III: 1 II: 2 I: 3 | IV: 9-10 points III: 11-13 points:  II:14-16 points I: 17-18 points |
| Median survival (months) original study | III: 2,3 II: 4,2  I: 7,1 | IV: 2,6 III: 3,8 II: 6,9 I: 11,0 | III: 2.91 II: 7.00 I: 31.38 | IV: 1,9 III: 3,3 II: 13,1 I: >32 | 6 month OS: IV: 6% III: 15% II: 43% I: 76% |

**Supplementary table 2: Odds ratios for receiving SRS before and after propensity score matching (PSM)**

|  | **before PSM (n=274)** | | | | **after PSM (n=168)** | | | |
| --- | --- | --- | --- | --- | --- | --- | --- | --- |
|  | OR | 95% CI for OR | | Sig. | OR | 95% CI for OR | | Sig. |
|  |  | Lower | Upper |  |  | Lower | Upper |  |
| Sex(male vs female) | 0.964 | 0.541 | 1.717 | 0.901 | 1.089 | 0.548 | 2.163 | 0.808 |
| primary tumor (NSCLC adeno vs Mamma) | 0.949 | 0.350 | 2.576 | 0.918 | 0.818 | 0.253 | 2.643 | 0.737 |
| primary tumor (NSCLC Adeno vs other) | 1.641 | 0.922 | 2.920 | 0.092 | 0.994 | 0.497 | 1.988 | 0.987 |
| number of CNS metastases | 1.356 | 0.946 | 1.944 | 0.633 | 1.001 | 0.993 | 1.010 | 0.776 |
| Time to CNS metastasis | 1.001 | 0.996 | 1.007 | 0.011 | 1.019 | 0.914 | 1.135 | 0.734 |
| PTV volume | 0.907 | 0.842 | 0.978 | 0.578 | 1.003 | 0.975 | 1.032 | 0.836 |
| Age | 0.993 | 0.969 | 1.017 | 0.738 | 1.033 | 0.819 | 1.302 | 0.785 |
| KPI | 0.966 | 0.787 | 1.185 | 0.579 | 1.122 | 0.488 | 2.576 | 0.787 |
| Extracerebral metastases (not present vs present) | 1.221 | 0.604 | 2.468 | 0.982 | 0.909 | 0.441 | 1.875 | 0.796 |
| systemic disease status (controlled vs progressive) | 1.007 | 0.546 | 1.856 | 0.985 | 1.089 | 0.548 | 2.163 | 0.808 |

**Supplementary table 3: Distribution of prognostic factors between female and male patients reported as odds ratios (95% CI)**

|  | **Before PSM (n=240)** | | | | **After PSM (n=164)** | | | |
| --- | --- | --- | --- | --- | --- | --- | --- | --- |
|  | OR | 95% CI for OR | | Sig. | OR | 95% CI for OR | | Sig. |
|  |  | Lower | Upper |  |  | Lower | Upper |  |
| primary tumor  (NSCLC adenoca. vs other) | 0.723 | 0.414 | 1.262 | 0.254 | 1.067 | 0.558 | 2.042 | 0.844 |
| number of CNS metastases | 1.128 | 0.781 | 1.628 | 0.521 | 1.002 | 0.652 | 1.541 | 0.992 |
| time to CNS metastasis | 0.999 | 0.992 | 1.006 | 0.783 | 1.000 | 0.991 | 1.008 | 0.975 |
| PTV volume | 1.013 | 0.957 | 1.071 | 0.663 | 0.997 | 0.921 | 1.080 | 0.951 |
| age | 0.979 | 0.955 | 1.003 | 0.091 | 1.007 | 0.976 | 1.039 | 0.672 |
| KPI | 1.064 | 0.859 | 1.317 | 0.571 | 0.994 | 0.770 | 1.282 | 0.962 |
| extracerebral metastases (not present vs present) | 1.127 | 0.559 | 2.275 | 0.738 | 0.889 | 0.383 | 2.063 | 0.783 |
| systemic disease status (controlled vs progressive) | 0.748 | 0.401 | 1.396 | 0.362 | 0.975 | 0.464 | 2.049 | 0.947 |

**Supplementary table 4: Cox models for each prognostic score using (training dataset)**

| N = 132 | category | HR | HR 95% CI | |
| --- | --- | --- | --- | --- |
|  |  |  | LB | UB |
| BSBM | 0 (reference) |  |  |  |
|  | 1 | 0.40** | 0.23 | 0.67 |
|  | 2 | 0.23*** | 0.12 | 0.42 |
|  | 3 | 0.18*** | 0.09 | 0.37 |
| Rades et al. | 0 (reference) |  |  |  |
|  | 1 | 0.34* | 0.11 | 1.00 |
|  | 2 | 0.37 | 0.13 | 1.04 |
|  | 3 | 0.18** | 0.06 | 0.55 |
| RPA | 1 (reference) |  |  |  |
|  | 2 | 2.37* | 1.18 | 4.76 |
|  | 3 | 5.51** | 1.99 | 15.25 |
| SIR | Poor (reference) |  |  |  |
|  | Intermediate | 0.65 | 0.09 | 4.67 |
|  | Good | 0.22 | 0.03 | 1.72 |
| GPA | 0 (reference) |  |  |  |
|  | 1 | 0.34* | 0.14 | 0.87 |
|  | 2 | 0.28* | 0.09 | 0.84 |
|  | 3 | 0.08*** | 0.02 | 0.34 |
| * *p* < 0.05, ** *p* < 0.01, *** *p* < 0.001 | | | | |
